# Incidence and risk factors of deep vein thrombosis in patients with spinal cord injury: a systematic review with meta-analysis

**DOI:** 10.3389/fcvm.2023.1153432

**Published:** 2023-05-12

**Authors:** Zhizhong Shang, Pingping Wanyan, Baolin Zhang, Mingchuan Wang, Xin Wang

**Affiliations:** ^1^ The First Clinical Medical College of Lanzhou University, Lanzhou, China; ^2^ Department of Pathology and Pathophysiology, Gansu University of Chinese Medicine, Lanzhou, China; ^3^ Department of Nephrology, The Second Hospital of Lanzhou University, Lanzhou, China; ^4^ Chengren Institute of Traditional Chinese Medicine, Lanzhou, China; ^5^ Department of Spine, Changzheng Hospital, Naval Medical University, Shanghai, China

**Keywords:** spinal cord injury, deep vein thrombosis, incidence, risk factor, systematic review

## Abstract

**Background:**

Spinal cord injury (SCI) is a highly disabling disease with huge public health burden. The complications associated with it, especially deep vein thrombosis (DVT), further aggravate the disability.

**Objective:**

To explore the incidence and risk factors of DVT after SCI, in order to provide guidance for disease prevention in the future.

**Methods:**

A search was performed on PubMed, Web of Science, Embase, and Cochrane database up to November 9, 2022. Literature screening, information extraction and quality evaluation were performed by two researchers. The data was later combined by metaprop and metan commands in STATA 16.0.

**Results:**

A total of 101 articles were included, including 223,221 patients. Meta-analysis showed that the overall incidence of DVT was 9.3% (95% CI: 8.2%–10.6%), and the incidence of DVT in patients with acute and chronic SCI was 10.9% (95% CI: 8.7%–13.2%) and 5.3% (95% CI: 2.2%–9.7%), respectively. The incidence of DVT decreased gradually with the accumulation of publication years and sample size. However, the annual incidence of DVT has increased since 2017. There are 24 kinds of risk factors that may contribute to the formation of DVT, involving multiple aspects of the baseline characteristics of the patient, biochemical indicators, severity of SCI, and comorbidities.

**Conclusions:**

The incidence of DVT after SCI is high and has been gradually increasing in recent years. Moreover, there are numerous risk factors associated with DVT. Comprehensive preventive measures need to be taken as early as possible in the future.

**Systematic Review Registration:**

www.crd.york.ac.uk/prospero, identifier CRD42022377466.

## 1. Introduction

Spinal cord injury (SCI) is a potentially fatal central nervous system disorder, which can lead to permanent loss of motor, sensory and autonomic nervous system function. There are more than 27 million patients with SCI worldwide, with about 250,000–500,000 new cases each year, with a lifetime cost of $750,000–$3 million per patient (1–4). Due to complex pathophysiological changes, there is no effective treatment available. In addition to the burden of SCI itself, the related complications can also cause serious consequences and burden to patients (5, 6).

The main complications of SCI are pressure sores, urinary complications, respiratory complications, deep venous thrombosis (DVT), spasm, pain, osteoporosis, among which DVT is one of the most serious complications (5, 7, 8). DVT refers to the abnormal coagulation of blood in the deep venous lumen, blocking the venous lumen, resulting in venous reflux obstruction, more common in the lower extremities. Patients with SCI have the highest risk of DVT among all hospitalized patients, up to 100% (9). After the formation of DVT in patients with SCI, only a few of the thromboses are confined to the site of occurrence or disappear on their own, and most of them extend to the trunk of the deep vein of the whole limb, which can cause a variety of complications, including post-thrombotic syndrome, edema, compression ulcer, recurrence of DVT and even pulmonary thromboembolism (10–12). Pulmonary thromboembolism caused by DVT can cause arrhythmia, hypoxia and rapid death, which are the third leading cause of death in SCI (13, 14). As a result, the prognosis for DVT is poor, with mortality rates of 20% at 2 years and up to 31% at 8 years (15).

Although there are obvious clinical manifestations such as muscle pain in the early stage of DVT, the chief complaint of patients is not obvious due to the partial or complete loss of superficial sensation after SCI. 50%–80% of patients with DVT had no obvious clinical symptoms or lack of specificity in the early stage, and more than 50% of the patients had normal physical examination, resulting in a high rate of missed diagnoses (16). The reported incidence of DVT after SCI ranges from 9% to 100% (9, 17). There are significant differences in the epidemiological results of DVT in patients with SCI reported in different studies, indicating that there is a lack of multicenter, large sample epidemiological data, and the risk factors for DVT are not clear (18, 19). In this pioneer study, we will systematically search for studies on DVT after SCI, comprehensively explore the incidence of DVT after SCI, and further explore its risk factors. It is very important for clinicians and patients to improve their awareness of DVT and to take preventive strategies as soon as possible.

## 2. Methods

This study complies with the PRISMA 2020 statement, the protocol was designed based on population, interventions, comparisons, outcomes, and study design (PICOS) criteria, and the protocol is registered (CRD42022377466) on PROSPERO (www.crd.york.ac.uk/prospero).

### 2.1. Inclusion and exclusion criteria

#### 2.1.1. Patients (P)

Spinal cord injury.

#### 2.1.2. Interventions (I)

Not applicable.

#### 2.1.3. Control (C)

Not applicable.

#### 2.1.4. Outcome (O)

Incidence and risk factors of DVT.

#### 2.1.5. Type of study (S)

Observational and experimental studies.

#### 2.1.6. Exclusion criteria

(1) Studies not meeting the criteria for PICOS. (2) Studies with small sample sizes (<30 for a single group). (3) Studies with incomplete data or obvious errors. (4) Patients with other serious diseases that may lead to DVT.

### 2.2. Databases search

PubMed, Web of science, Embase, and Cochrane database was searched by computer from the establishment of the database to November 9, 2022. The retrieval adopts the combination of subject words and free words. At the same time, the references included in the study were searched to supplement and obtain the relevant data. The search terms include: (thrombosis OR thrombus OR blood clots OR thromboembolism OR phlebothrombosis venous thrombosis OR phlebothrombos OR venous thrombos OR vein thrombosis OR deep vein thrombosis OR deep venous thrombosis OR lower extremity deep vein thrombosis OR deep venous thrombosis of lower limb OR venous thromboembolism OR deep vein thrombos OR deep venous thrombos) AND (Spinal cord injury OR Spinal injury OR Spinal Cord Trauma OR Spinal Cord Transection OR Spinal Cord Laceration OR Post-Traumatic Myelopathy OR Spinal Cord Contusion). For more search details about each database, see Table 1.

**Table 1:** Search strategies.

**1. PubMed** #1: "Thrombosis"[MeSH Terms] OR "Thromboembolism"[MeSH Terms] OR "Venous Thromboembolism"[MeSH Terms] OR "Venous Thrombosis"[MeSH Terms] 193,259 #2: "thrombosis"[Title/Abstract] OR "thrombus"[Title/Abstract] OR "blood clots"[Title/Abstract] OR "thromboembolism"[Title/Abstract] OR (("venous thrombosis"[MeSH Terms] OR ("venous"[All Fields] AND "thrombosis"[All Fields]) OR "venous thrombosis"[All Fields] OR "phlebothrombosis"[All Fields]) AND "venous thrombosis"[Title/Abstract]) OR (("veins"[MeSH Terms] OR "veins"[All Fields] OR "venous"[All Fields]) AND "thrombos"[Title/Abstract]) OR "vein thrombosis"[Title/Abstract] OR "deep vein thrombosis"[Title/Abstract] OR "deep venous thrombosis"[Title/Abstract] OR "lower extremity deep vein thrombosis"[Title/Abstract] OR "deep venous thrombosis of lower limb"[Title/Abstract] OR "venous thromboembolism"[Title/Abstract] OR "deep vein thrombos"[Title/Abstract] OR (("deep"[All Fields] AND ("veins"[MeSH Terms] OR "veins"[All Fields] OR "venous"[All Fields])) AND "thrombos"[Title/Abstract]) 222,879 #3: #1 OR #2 300,452 #4: "spinal cord injury"[Title/Abstract] OR "spinal injury"[Title/Abstract] OR "spinal cord trauma"[Title/Abstract] OR "spinal cord transection"[Title/Abstract] OR "spinal cord laceration"[Title/Abstract] OR "post traumatic myelopathy"[Title/Abstract] OR "spinal cord contusion"[Title/Abstract] 45,122 #5: "Spinal Cord Injuries"[MeSH Terms] OR "Spinal Injuries"[MeSH Terms] 77,498 #6: #4 OR #5 90,939 #7: #3 AND #6 875 **2. WOS** (TS=(thrombosis OR thrombus OR blood clots OR thromboembolism OR phlebothrombosis venous thrombosis OR phlebothrombos OR venous thrombos OR vein thrombosis OR deep vein thrombosis OR deep venous thrombosis OR lower extremity deep vein thrombosis OR deep venous thrombosis of lower limb OR venous thromboembolism OR deep vein thrombos OR deep venous thrombos)) AND TS=(Spinal cord injury OR Spinal injury OR Spinal Cord Trauma OR Spinal Cord Transection OR Spinal Cord Laceration OR Post-Traumatic Myelopathy OR Spinal Cord Contusion) 1846 **3. Embase** #1: 'thrombosis'/exp 438,851 #2: 'thrombus'/exp 30,056 #3: 'thromboembolism'/exp 640,440 #4: 'vein thrombosis'/exp 158,927 #5: 'deep vein thrombosis'/exp 76,503 #6: ' 'deep venous thrombosis'/exp 76,503 #7: 'lower extremity deep vein thrombosis'/exp 2,263 #8: 'venous thromboembolism'/exp 192,144 #9: #1 OR #2 OR #3 OR #4 OR #5 OR #6 OR #7 OR #8 704,194 #10: 'spinal cord injury':ab,ti OR 'spinal injury':ab,ti OR 'spinal cord trauma':ab,ti OR 'spinal cord transection':ab,ti OR 'spinal cord laceration':ab,ti OR 'post-traumatic myelopathy':ab,ti OR 'spinal cord contusion':ab,ti 57,045 #11: 'spinal cord injury'/exp 90,227 #12: 'spinal injury'/exp 66,366 #13: 'spinal cord trauma'/exp 90,227 #14: 'spinal cord transection'/exp 3,334 #15: 'spinal cord contusion'/exp 76 #16: #10 OR #11 OR #12 OR #13 OR #14 OR #15 #17: #9 AND #16 AND ([article]/lim OR [article in press]/lim) 2763 **4. Cochrane** #1: MeSH descriptor: [Thrombosis] explode all trees 5119 #2: MeSH descriptor: [Blood Coagulation] in all MeSH products 2433 #3: MeSH descriptor: [Venous Thrombosis] explode all trees 2859 #4: MeSH descriptor: [Venous Thromboembolism] explode all trees 805 #5: (thrombosis OR thrombus OR blood clots OR thromboembolism OR phlebothrombosis venous thrombosis OR phlebothrombos OR venous thrombos OR vein thrombosis OR deep vein thrombosis OR deep venous thrombosis OR lower extremity deep vein thrombosis OR deep venous thrombosis of lower limb OR venous thromboembolism OR deep vein thrombos OR deep venous thrombos):ti,ab,kw 34652 #6: #1 OR #2 OR #3 OR #4 OR #5 36661 #7: MeSH descriptor: [Spinal Cord Injuries] explode all trees 1956 #8: MeSH descriptor: [Spinal Injuries] explode all trees 895 #9: #7 or #8 2828 #10: #6 and #9 43

### 2.3. Article selection and information extraction, and quality assessment

Two reviewers conducted the initial screening of the literature according to the title and abstract, and then obtained the full text for re-screening to determine the studies that met the inclusion criteria. In case of disagreement, a third reviewer is requested to assist in the judgment. Finally, the author, year of publication, country, type of study, age, sample size, male/female ratio, site of SCI, American Spinal Injury Association Impairment Scale (AIS), injury time, treatment measures, diagnostic methods, incidence, or number of DVT and influencing factors were extracted from the final included literature. In addition, methodological index for non-randomized study (MINORS) was used to assess the quality of evidence included in the study (20). MINORS consists of 12 items, each with a score of 0 (not reported), 1 (reported but inadequate), or 2 (reported and adequate). The full score is 24.

### 2.4. Statistical analysis

The incidence of DVT was calculated by using the metaprop command of STATA16.0 software, and the results were expressed in terms of incidence and 95% confidence interval (CI). The metan command was used to calculate the risk factors of DVT, and the results were expressed by adjusted odds ratio (AOR) and its 95% CI. If there was no statistical heterogeneity among the studies (*P* < 0.05 and *I*^2^ < 50%), the fixed effect model was used for meta-analysis. Otherwise, the source of heterogeneity was further analyzed, and the random effects model was used for meta-analysis. Funnel chart was drawn to detect publication bias.

## 3. Results

### 3.1. Literature search and screening

**Figure 1:**
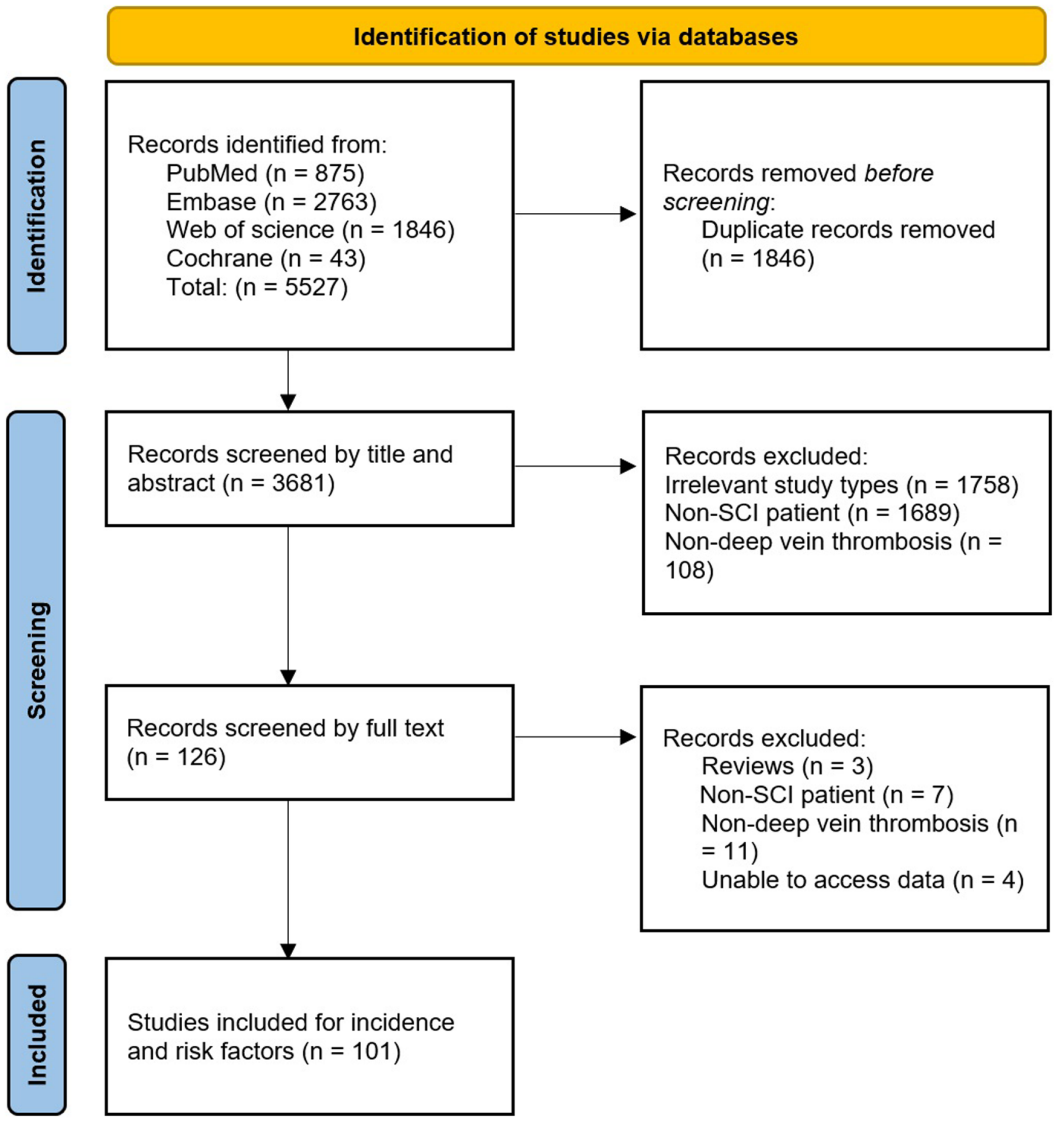
The PRISMA flow chart.

A total of 5,527 records were obtained from four databases. After excluding duplicate records that did not meet the inclusion criteria, 101 studies were included. These studies all reported the incidence of DVT, of which 12 reported risk factors for DVT (Figure 1).

### 3.2. Basic information of included studies

The 101 studies included 75 cohort studies, 16 cross-sectional studies, 5 case-control studies and 5 experimental studies. The age of the patients varied widely, most of them were between 30 and 60 years old, and 16 studies did not report the age of the patients. The sample size of a single study is between 37 and 78,016, and the total sample size is 223,221. The severity of SCI varied from AIS grades A–E, and no AIS score was reported in 35 studies. Sites of injury included the cervical, thoracic, and lumbar cord, and 16 studies did not report sites of injury. 53 studies included patients with acute SCI, 6 studies included patients with chronic SCI, 14 studies did not limit the timing of SCI, and 28 studies did not report the timing of SCI. DVT was diagnosed by doppler ultrasound. For more details about basic information, see Table 2.

**Table 2:** Basic information of the included studies.

Author	Year	Country	Study type	Age	Sample	M/F	Severity	Injury site	Timings	Diagnosis method
Thumbikat	2002	England	Cross-sectional study	/	173	129/44	/	Unlimited	Acute	Doppler ultrasound
Bai	2021	China	Experimental research	38.45 ± 12.23/39.27 ± 11.19	80	47/33	A–C	Cervical	/	Doppler ultrasound
lv	2022	China	Cross-sectional study	58.7 ± 11.9/52.6 ± 13.5	175	129/46	/	Cervical	Acute	Doppler ultrasound
Yu	2020	China	Cross-sectional study	47.77 ± 10.76/37.35 ± 10.20	80	36/44	/	Thoracolumbar	/	Doppler ultrasound
Chen	2016	China	Cross-sectional study	48 (35–57)	377	184/193	A–D	Unlimited	Acute	Doppler ultrasound
Joseph	2017	South Africa	Cohort study	26 ± 6.9	145	96/49	A–D	Unlimited	Acute	Doppler ultrasound
Chiou-Tan	2003	USA	Experimental research	36.1 ± 15	95	72/23	/	Unlimited	Acute	Doppler ultrasound
McKinley	2002	USA	Cohort study	55.3 ± 13.6/38.5 ± 16.4	117	89/28	A–D	Unlimited	Acute	Doppler ultrasound
Aito	2003	Italy	Cohort study	38.9	588	476/112	A–E	Unlimited	Chronic	Doppler ultrasound
Tauqir	2007	Pakistan	Cross-sectional study	16–39	194	50/144	A–E	Unlimited	Acute	Doppler ultrasound
Roussi	1999	France	Cohort study	17–72	67	52/15	/	Unlimited	Acute	Doppler ultrasound
Cho	2020	Korea	Cohort study	60.47 ± 17.41	45	/	/	Unlimited	/	Doppler ultrasound
Wu	2021	China	Cohort study	53.1 ± 16.2	1,290	1,130/160	A–D	Cervical	Acute	Doppler ultrasound
Anbesaw	2001	USA	Cohort study	45.5 ± 20.2	3,389	2,542/847	A–D	Unlimited	Acute	Doppler ultrasound
Hebbeler	2017	USA	Cohort study	/	129	/	/	Unlimited	Acute	Doppler ultrasound
Worley	2008	Canada	Cohort study	46.0 ± 19.9/38.0 ± 16.4	90	79/11	A–E	Unlimited	Acute	Doppler ultrasound
Kumagai	2018	Japan	Cohort study	59.7 ± 18.8/65.4 ± 15.2	113	84/30	A–E	Unlimited	Acute	Doppler ultrasound
Masuda	2015	Japan	Cohort study	61.3 (13–91)	268	223/45	A–E	Cervical	Acute	Doppler ultrasound
Du	2018	China	Cohort study	43.4 ± 13.9/45.1 ± 14.2	721	502/219	B–D	Thoracolumbar	Acute	Doppler ultrasound
Riklin	2003	Switzerland	Cohort study	46.6 (5–90)	1,209	887/322	/	Unlimited	/	Doppler ultrasound
Agarwal	2009	India	Cohort study	32 (10–80)	297	241/56	A–E	Unlimited	Acute	Doppler ultrasound
Yao	2009	Sugimoto	Cohort study	/	52	41/11	A–D	Cervical	Acute	Doppler ultrasound
Mackiewicz	2016	Poland	Cross-sectional study	32 (13–65)	53	48/15	A–D	Unlimited	Chronic	Doppler ultrasound
Groves	2017	Nepal	Cross-sectional study	30 ± 16.4	117	66/51	A–E	Unlimited	/	Doppler ultrasound
Sara	2015	Canada	Cohort study	51.6 ± 21.3/53.8 ± 18.6	7,693	4,083/3,610	A–E	Unlimited	/	Doppler ultrasound
Germing	2010	Germany	Cohort study	19–90	139	88/51	/	/	/	Doppler ultrasound
Powell	1999	USA	Case–control study	44 (13–91)	189	144/45	A–D	Unlimited	Chronic	Doppler ultrasound
Hon	2019	USA	Cohort study	51.2 ± 20.2	189	140/49	A–D	Unlimited	Acute	Doppler ultrasound
Chang	2017	Houston	Cohort study	28–62	501	364/137	A–D	Unlimited	/	Doppler ultrasound
Cao	2019	China	Cohort study	21–58	342	213/129	A–D	Unlimited	/	Doppler ultrasound
Mackiewicz	2020	Poland	Cohort study	37.7 ± 15.7	78	66/12	A–C	Unlimited	/	Doppler ultrasound
Waters	1999	USA	Case–control study	/	3,756	/	/	/	Acute	Doppler ultrasound
Christina	2012	USA	Cohort study	/	140	97/43	A–D	/	Acute	Doppler ultrasound
Nwankwo	2013	Nigeria	Cohort study	2–75	85	69/16	A–E	Unlimited	/	Doppler ultrasound
Wang	2020	China	Cross-sectional study	39.5 ± 11.2	3,487	2,509/978	A–D	Unlimited	/	Doppler ultrasound
Du	2020	China	Cohort study	50.1 ± 14.8	1,730	1,284/446	A–D	Unlimited	/	Doppler ultrasound
Rathore	2007	Pakistan	Cohort study	28.3 ± 12.4	187	80/107	A–E	Unlimited	Acute	Doppler ultrasound
Wu	2012	China	Cohort study	54.6 ± 14.6	143	119/24	A–D	Unlimited	/	Doppler ultrasound
Li	2012	China	Cohort study	38.5 ± 9.6	51	21/30	A–D	Unlimited	/	Doppler ultrasound
Green	2005	USA	Cohort study	44.5 ± 19.1	76	55/21	/	/	/	Doppler ultrasound
Wu	2013	China	Cohort study	>15	631	537/94	A–D	Unlimited	Unlimited	Doppler ultrasound
Mackiewicz	2021	Poland	Cross-sectional study	36.4	88	75/13	A–C	Unlimited	Unlimited	Doppler ultrasound
Ichikawa	2020	Japna	Cohort study	68.4 ± 12.8/63.7 ± 18.5	57	44/13	A–E	Cervical	Acute	Doppler ultrasound
Do	2013	Korea	Cohort study	49.1 ± 16.5	185	133/52	A–D	Unlimited	/	Doppler ultrasound
Chung	2014	China	Cohort study	50 ± 19.9	47,916	30,036/17,880	A–D	Unlimited	Unlimited	Doppler ultrasound
Geoffrey	1993	USA	Cohort study	22-81	287	282/5	/	/	Chronic	Doppler ultrasound
William	1999	USA	Cross-sectional study	/	6776	/	A–E	Unlimited	Unlimited	Doppler ultrasound
Chen	1999	USA	Cross-sectional study	36.5 ± 16.9	1,649	/	A–E	Unlimited	Unlimited	Doppler ultrasound
Carbone	2013	USA	Case–control study	21.49–94.37	2,054	2,054/0	A–E	Unlimited	Chronic	Doppler ultrasound
Arsh	2019	Pakistan	Cohort study	41.7 ± 17.3	62	46/16	/	/	/	Doppler ultrasound
Cho	2021	Korea	Cohort study	/	167	/	/	/	Acute	Doppler ultrasound
McKinley	2004	USA	Cohort study	37.65 ± 15.83	779	614/165	A–E	Unlimited	Acute	Doppler ultrasound
Halim	2014	India	Experimental research	14–66	74	60/14	A–D	Unlimited	Acute	Doppler ultrasound
Scivoletto	2018	Italy	Cohort study	40.6 ± 17.3	250	209/41	A–D	Unlimited	Acute	Doppler ultrasound
Joseph	2015	Sweden	Cohort study	33.5 ± 13.8	145	124/21	/	Unlimited	Acute	Doppler ultrasound
SCTI	2003	USA	Experimental research	36.9 ± 16.7	476	389/87	A–D	Cervical thoracic	/	Doppler ultrasound
Marion	2016	Canada	Cohort study	47.5 ± 20.3	444	352/92	A–D	Unlimited	/	Doppler ultrasound
Gorman	2008	USA	Cohort study	48.1 ± 19.7	58	40/18	/	Unlimited	Acute	Doppler ultrasound
FRCS	1992	England	Cohort study	/	100	80/20	/	Unlimited	/	Doppler ultrasound
Deep	2001	England	Cohort study	/	130	/	/	Unlimited	Acute	Doppler ultrasound
Singh	2022	India	Cohort study	33.38 ± 12.97	50	40/10	A–E	Unlimited	Acute	Doppler ultrasound
Citak	2012	Germany	Case–control study	/	264	212/52	/	Unlimited	Acute	Doppler ultrasound
Eichinger	2018	Austria	Cohort study	47.8 ± 18.3	185	162/23	A–D	Unlimited	/	Doppler ultrasound
Maxwell	2002	USA	Cohort study	/	111	90/21	/	/	Acute	Doppler ultrasound
Sasa	2012	Serbia	Cohort study	>18	441	322/119	/	/	/	Doppler ultrasound
Hetz	2011	Canada	Cross-sectional study	46.83 ± 13.42	695	531/164	A–D	Unlimited	Chronic	Doppler ultrasound
Noreau	2000	Canada	Cross-sectional study	42.4 ± 12.1	482	392/90	A–D	Unlimited	Unlimited	Doppler ultrasound
Germing	2009	Germany	Cross-sectional study	19-85	115	75/40	/	Unlimited	Acute	Doppler ultrasound
Ametefe	2016	Ghana	Cohort study	36.25 ± 13.62	185	141/44	/	Unlimited	Unlimited	Doppler ultrasound
Schottler	2012	USA	Cohort study	11	159	92/67	/	Unlimited	Unlimited	Doppler ultrasound
Jentzsch	2021	Canada	Cohort study	46	460	86/361	/	Unlimited	Acute	Doppler ultrasound
Kadyan	2003	USA	Cohort study	15–86	92	68/24	A–D	Unlimited	Unlimited	Doppler ultrasound
Yelnik	1991	France	Cohort study	34.5 ± 15.5	147	106/41	/	Unlimited	Acute	Doppler ultrasound
Colachis	1993	USA	Cohort study	/	209	/	/	/	Acute	Doppler ultrasound
Maharaj	2016	Australia	Cohort study	41 ± 18	384	328/56	A–D	Unlimited	Acute	Doppler ultrasound
Furaln	2005	Canada	Cohort study	17–89	55	38/17	A–D	Cervical	Acute	Doppler ultrasound
Rathore	2008	Pakistan	Cohort study	28.3 ± 12.4	187	80/107	A–D	Unlimited	Acute	Doppler ultrasound
Chen	2012	China	Experimental research	42.11 ± 13.75	295	183/112	A–D	Cervical	Acute	Doppler ultrasound
Hao	2021	China	Cohort study	/	78,061	/	A–E	Unlimited	Unlimited	Doppler ultrasound
Mackiewicz	2021	Poland	Cohort study	37.3 ± 15.7	145	115/30	A–C	Unlimited	Acute	Doppler ultrasound
Lau	2019	USA	Cohort study	52.9 ± 19.4	106	77/29	A–D	Unlimited	Acute	Doppler ultrasound
Jones	2005	USA	Cohort study	44.5 ± 21	16,240	11,777/4,463	A–D	Unlimited	Unlimited	Doppler ultrasound
Ahlquist	2020	USA	Cohort study	>18	79	61/18	A–D	Unlimited	Acute	Doppler ultrasound
Saraf	2007	India	Cohort study	16–59	70	/	/	Unlimited	Acute	Doppler ultrasound
Morita	2018	Japan	Cohort study	14–73	75	43/32	A–E	Thoracolumbar	Acute	Doppler ultrasound
Godat	2022	USA	Cohort study	18–40	343	/	/	Unlimited	Acute	Doppler ultrasound
Waring	1991	USA	Cohort study	33 ± 0.4	1419	1,149/270	A–D	Unlimited	Acute	Doppler ultrasound
Wang	2016	China	Cohort study	55 (43–62)	279	21/147	A–D	Unlimited	Acute	Doppler ultrasound
Zhang	2022	China	Cross-sectional study	/	260	193/67	A–D	Unlimited	/	Doppler ultrasound
Wu	2019	China	Cross-sectional study	34–66	207	155/52	A–D	Unlimited	Acute	Doppler ultrasound
Hoh	2015	USA	Cohort study	47.7 ± 22.3	10,669	7,761/2,908	A–E	Unlimited	Unlimited	Doppler ultrasound
Clements	2016	Australia	Cohort study	44 (25–61)	222	174/48	A–E	Unlimited	Acute	Doppler ultrasound
Guerra	2014	Brazil	Cohort study	20–76	100	72/28	A–D	Unlimited	/	Doppler ultrasound
Piran	2016	Canada	Cohort study	51 (17–91)	151	106/45	/	/	Acute	Doppler ultrasound
Chung	2011	Korea	Cohort study	53 ± 16.6	37	26/11	A–D	Unlimited	Acute	Doppler ultrasound
Lowery	2021	USA	Cohort study	44.6 ± 17.9	148	125/23	/	/	/	Doppler ultrasound
Winemiller	1999	USA	Case–control study	/	428	/	/	/	/	Doppler ultrasound
Maung	2011	USA	Cohort study	/	18,302	/	A–E	Unlimited	Unlimited	Doppler ultrasound
DiGiorgio	2017	USA	Cohort study	53.5 (18–49)	49	32/17	/	/	Acute	Doppler ultrasound
Green	2003	USA	Cohort study	40.1 ± 18.4	243	193/50	/	/	/	Doppler ultrasound
Pierfranceschi	2013	Italy	Cohort study	40.3 ± 15.9	94	80/14	/	/	Unlimited	Doppler ultrasound

### 3.3. Methodological quality assessment

**Figure 2:**
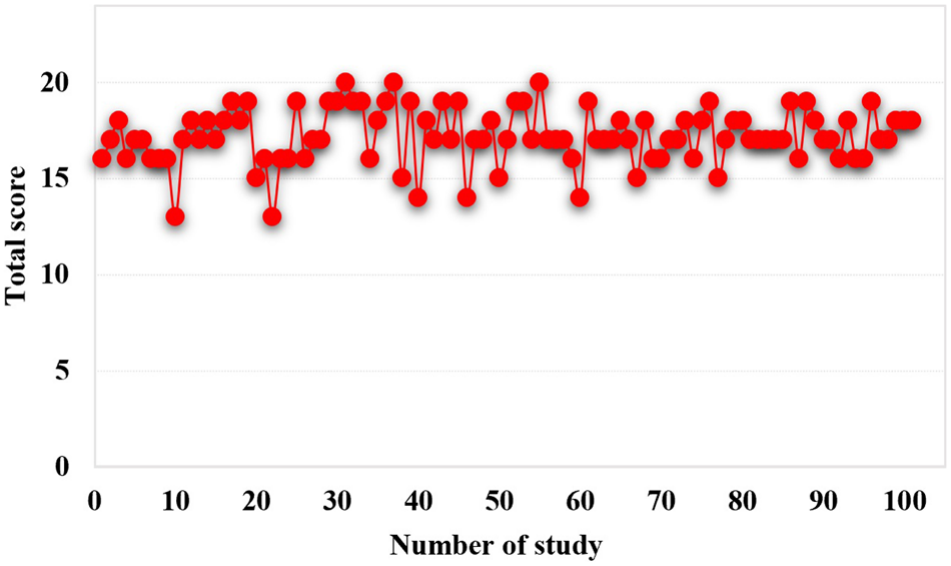
Score of quality evaluation.

The 101 studies included clearly reported the purpose of the study, 94 studies clearly reported the evaluation criteria of the results, 92 studies had appropriate control groups, 63 studies were balanced and comparable in baseline characteristics, and 97 studies conducted adequate data analysis. However, no study developed a protocol in advance, and no study prospectively calculated the required sample size. Only 4 studies blinded the evaluators of the results, only 21 studies had adequate follow-up of patients, and 57 studies had a loss of follow-up. Overall, out of a total of 24, only 41 studies scored 18 or more (Figure 2).

### 3.4. Meta-analysis results

#### 3.4.1. Incidence of DVT

**Figure 3:**
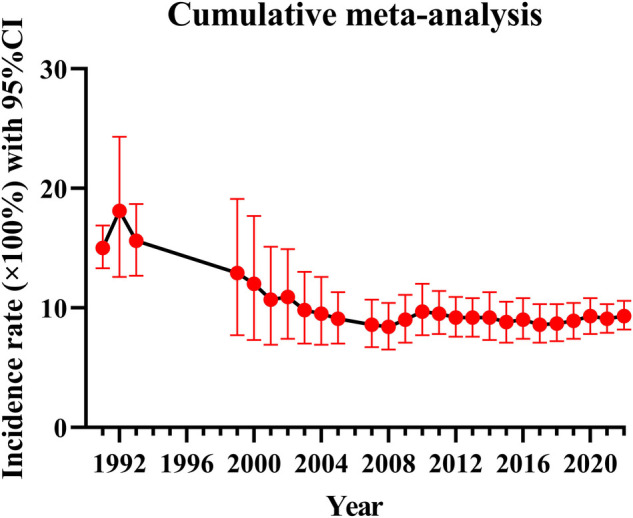
The incidence of DVT accumulated over the year.

**Figure 4:**
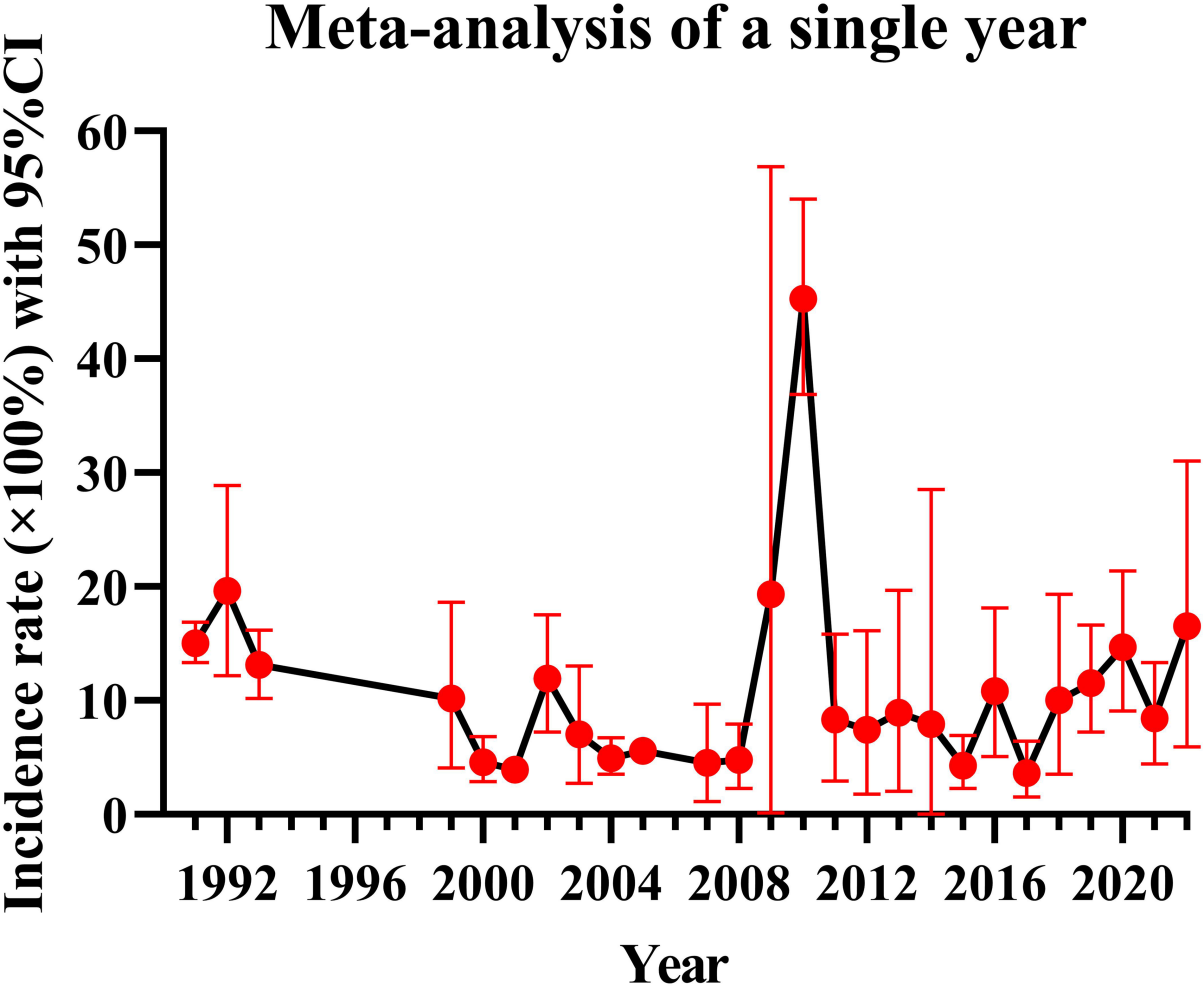
Annual DVT incidence.

**Figure 5:**
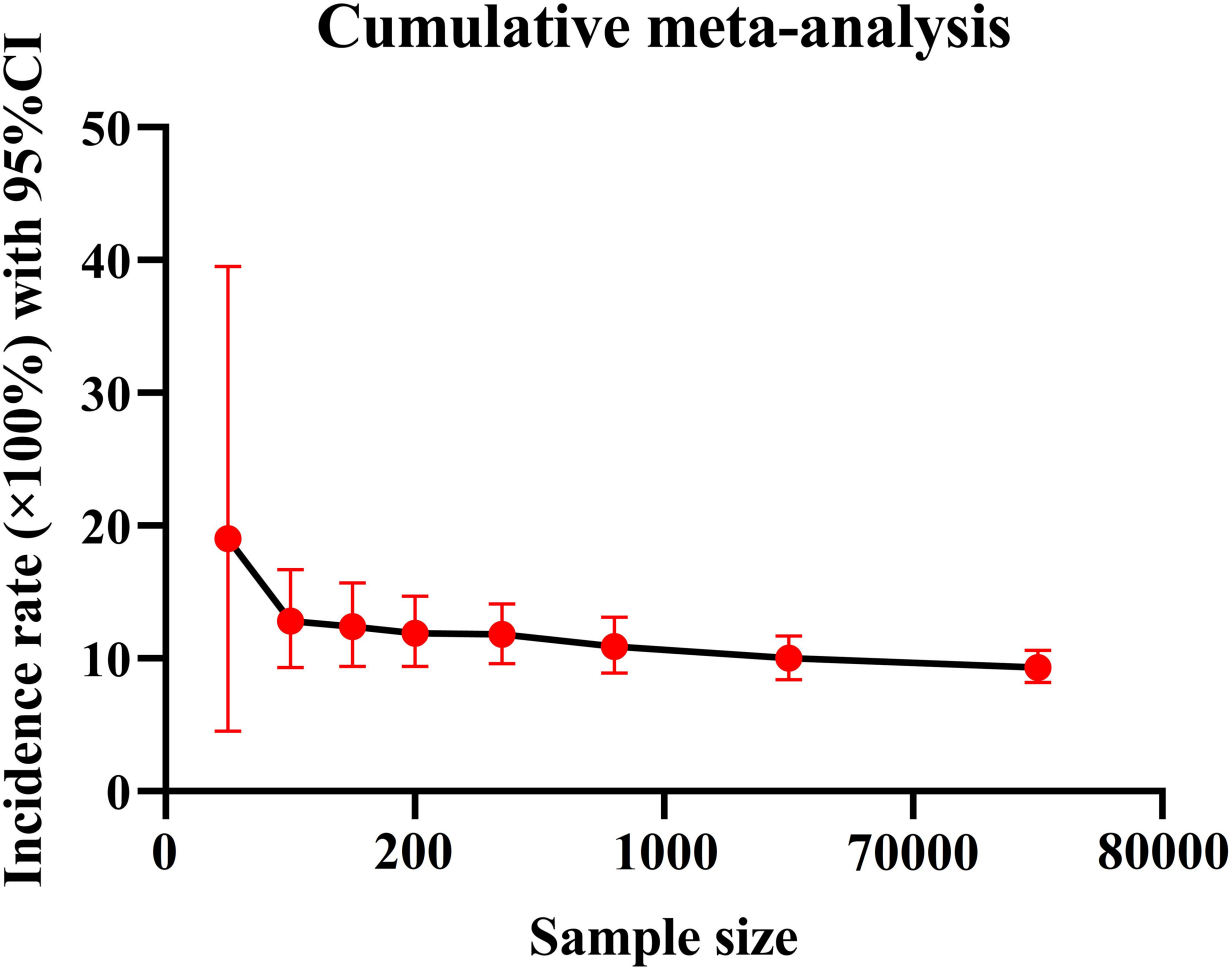
The incidence of DVT accumulated with the sample size.

Because of the great heterogeneity among different studies (*I*^2^ > 90%), the random effect model was used for Meta-analysis. The results showed that the overall incidence of DVT after SCI was 9.3% (95% CI: 8.2%–10.6%). The incidence of DVT in patients with acute and chronic SCI was 10.9% (95% CI: 8.7%–13.2%) and 5.3% (95% CI: 2.2%–9.7%). Since the incidence of DVT in patients with SCI was first reported in 1991, the overall incidence of DVT gradually decreased and tended to be stable with the accumulation of publication years (Figure 3). However, the incidence of DVT still varies greatly from year to year, and has a gradually increasing trend since 2017 (Figure 4). In addition, when the sample size of a single study gradually increased from 37 to 78,016, the cumulative incidence of DVT decreased with the increase of sample size (Figure 5).

#### 3.4.2. Risk factors for DVT

**Figure 6:**
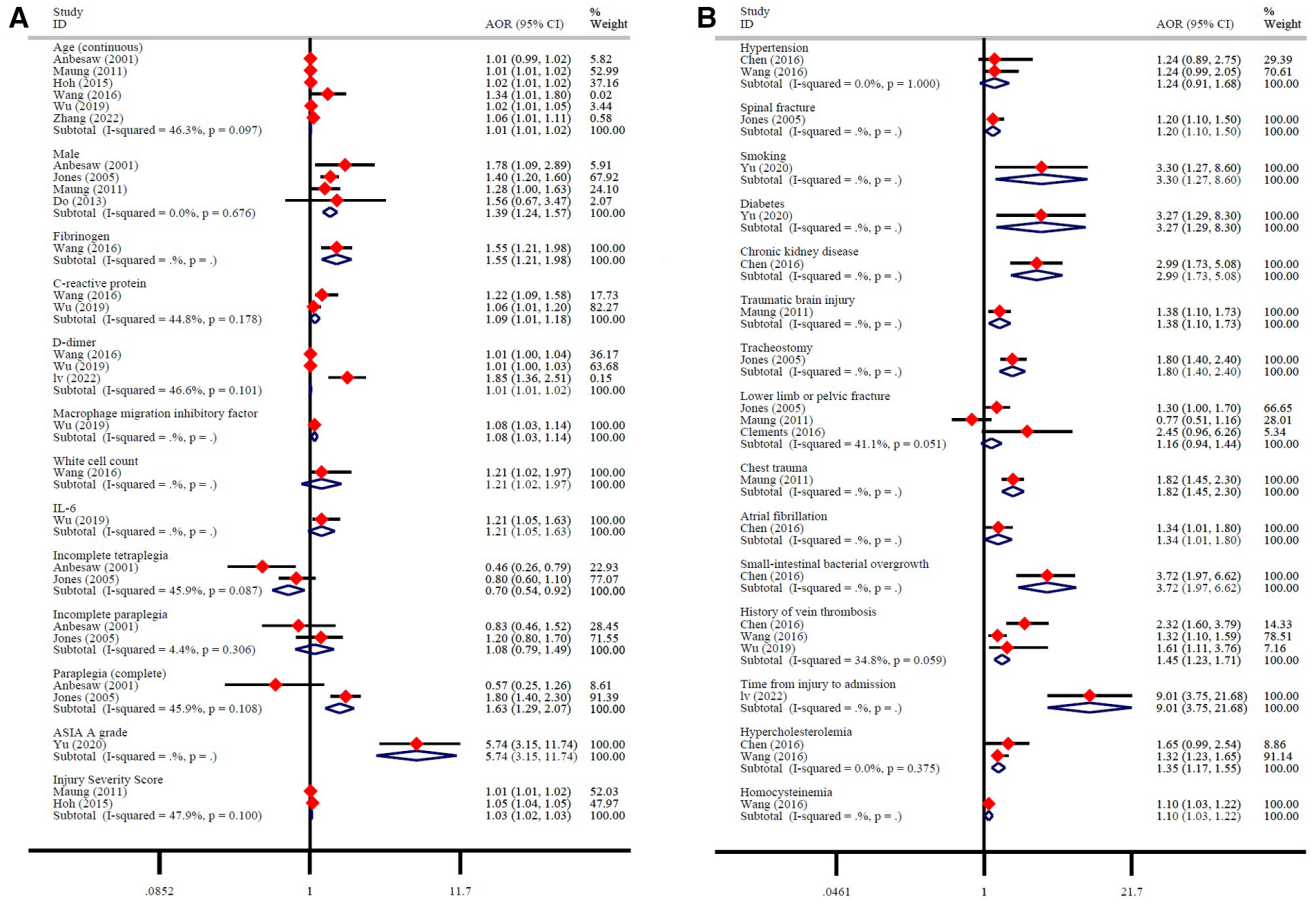
Risk factors for DVT.

A total of 12 studies reported 28 possible DVT-related factors. These influencing factors can be divided into three aspects. ① Baseline characteristics: Age is a risk factor for DVT. Men with SCI have a higher risk of developing DVT than women (Figure 6A). ② Biochemical indicators: Fibrinogen, C-reactive protein, D-dimer, macrophage migration inhibitory factor, white cell count, IL-6 are risk factors for DVT (Figure 6A). ③ Severity of SCI: AIS grade A, and injury severity score are risk factors for DVT. More specific, compared with tetraplegia, incomplete tetraplegia is the protective factor of DVT, there is no significant correlation between incomplete paraplegia and DVT, while complete paraplegia is the risk factor of DVT (Figure 6A). ④ Comorbidities: spinal fracture, smoking, diabetes, chronic kidney disease, traumatic brain injury, tracheostomy, chest trauma, atrial fibrillation, small-intestinal bacterial overgrowth, history of vein thrombosis, time from injury to admission, hypercholesterolemia, homocysteine are risk factors for DVT. There was no significant correlation between hypertension, lower limb or pelvic fracture and DVT (Figure 6B).

#### 3.4.3. Publication bias detection

**Figure 7:**
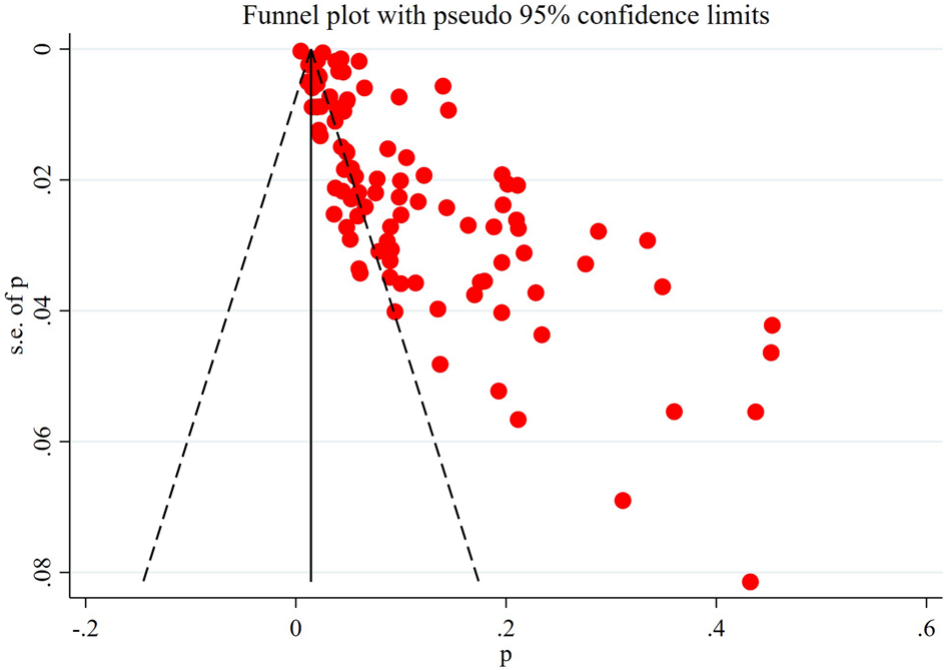
Funnel plot for publication bias.

The obviously asymmetric funnel plot showed that there was a greater possibility of publication bias in the current research field (Figure 7).

## 4. Discussion

### 4.1. Research overview

After SCI, the dysfunction of the autonomic nervous system leads to the dilatation of venous vessels of lower extremities and the increase of venous blood flow resistance (21, 22), the decrease of platelet inhibition of thrombin production and release of prostaglandins, the decrease of endothelial function, the enhancement of coagulation function and the relative weakening of fibrinolytic function, so that the blood is in a state of hypercoagulability (23, 24). In addition, due to various incentives such as SCI itself, limb paralysis after SCI, limb immobilization, long-term bed rest, surgery and anesthesia for anatomical reduction and maintaining spinal stability, it can favor the formation of DVT. Most studies do not pay enough attention to DVT after SCI, or do not comprehensively and systematically study the incidence and risk factors of DVT. For example, studies by Schottler et al. showed that DVT occurred in 3 of the 138 patients with acute SCI, with an incidence of only 2.2% (25). This is highly misleading, suggesting that the incidence of DVT is low and decreasing the attention of clinicians and patients. Although these data are based on realistic clinical data, the results from these small-sample, single-center studies are difficult to apply to the broader SCI population due to a variety of factors, including the health and disease status of the patients, the diagnostic criteria for DVT, and the design and sample size of the studies. Therefore, a summary analysis of all currently published studies to explore the incidence and risk factors of DVT in a comprehensive manner is essential for future clinical practice.

The incidence of DVT in patients with SCI reported in the previous literature varies greatly, ranging from 9% to 100% (9, 17). The reason for this disparity may be due to inconsistencies in the diagnostic methods of DVT, such as venography, doppler ultrasound, clinical manifestations or a combination of multiple examinations, and the small number of cases included in the analysis. Considering the above factors, we only included studies using doppler ultrasound to diagnose DVT according to the unified standard, and collected the data of 223,221 people in 101 studies, which reduced the influence of confounding factors to some extent. We also excluded studies that combined with other diseases that may lead to the formation of DVT and studies with a smaller sample size. Our study found that the overall incidence of DVT after SCI was 9.3% (95% CI: 8.2%–10.6%), and the incidence of DVT in patients with acute SCI was twice as high as that in patients with chronic SCI. This is consistent with the results of most studies. Most cases of DVT occur in the acute stage of SCI (26). When the sample size of a single study gradually increased from 37 to 78,016, the cumulative incidence of DVT decreased with the increase of sample size, indicating that the incidence of DVT was affected by the sample size. When the sample size is too small, the sporadic occurrence of DVT will greatly affect its incidence. This is also the main reason why we exclude small sample studies in advance. Of the 101 studies included, 5 studies had a sample size of more than 10,000 cases. In these five studies, the incidence of DVT ranged from 0.5% to 6%, lower than the overall incidence of 9.3%. Therefore, despite excluding small sample studies, the small sample effect may still affect the incidence of DVT. In addition, since the incidence of DVT in patients with SCI was first reported in 1991, the overall incidence of DVT gradually decreased with the accumulation of publication years and tended to be stable in 2010. In fact, the incidence of DVT still varies greatly from year to year. In particular, the incidence of DVT has been increasing in the past five years after it fell to the lowest point in 2017. The main reason is that in recent years, traffic accidents, falls and sports injuries have been increasing, resulting in an increase in the number of patients with SCI, and the number of DVT will naturally increase accordingly (27). In a nutshell, DVT cannot be ignored after SCI, and more large-scale study is needed in the future.

After defining the incidence of DVT in patients with SCI, it is very important to further explore the risk factors of DVT for its early prevention and treatment. This study shows that men have a higher risk of developing DVT than women. However, it is not clear why gender is an influencing factor of DVT. This may be related to the large difference in the proportion of men and women included in the study (76,715/35,925), or to the different probability of gender-induced exposure to risk factors. We also found that the older the age, the higher the incidence of DVT. With the increase of age, the various organs of the patient are in a state of decline, the blood viscosity increases, the blood flow is slow, the concentration of thrombotic factor in vascular endothelial cells increases, and antithrombin and other substances decrease, resulting in the destruction of coagulation-anticoagulation balance in the blood system, which leads to thrombosis (28). In this process, fibrinogen and D-dimer in blood are closely related to DVT, which are the risk factors of DVT (29). Fibrinogen is a coagulation factor synthesized and secreted by liver cells. When the human fibrinolytic system is activated, fibrinogen is converted into fibrin monomer and crosslinked with factor XIII, which is hydrolyzed into D-dimer induced by plasmin. When fibrinogen increases, the production of fibrinolysis and D-dimer increases, the blood is in a state of hypercoagulability. The blood viscosity increases, and the blood flow slows down, which can easily lead to thrombosis (30). In addition, inflammatory processes plays a key role in the formation of DVT by activating monocytes and endothelial cells, releasing cytokines and chemokines to participate in the activation of blood coagulation system, thus inducing hypercoagulable state (31). van Aken et al. found that the levels of interleukin-6, interleukin-8 and monocytechemotacticprotein1 (MCP-1) were significantly increased in patients with recurrent DVT (31). Ray et al. further found that IL-6 can increase anticoagulant factors such as tissue factor, fibrinogen and coagulation factor VIII, and reduce antithrombin and protein S, thus inducing hypercoagulable state and increasing the incidence of thrombotic diseases (32). Our results showed that inflammatory markers such as IL-6, C-reactive protein, macrophage migration inhibitory factor, and white cell count were significantly elevated in patients who developed DVT after SCI and were risk factors for DVT. In addition to inflammatory and coagulation indexes, hyperhomocysteinemia and hypercholesterolemia are also risk factors for DVT. Homocysteine is the intermediate product of methionine metabolism *in vivo*, which can directly activate V, X and XII factors, reduce the production of nitric oxide and prostacyclin, tissue plasminogen activator and adenosine diphosphatase, and increase the synthesis and expression of thromboxane A2 and P-selectin, thus promoting platelet adhesion, aggregation and thrombosis (33, 34). Cholesterol can also increase the production of coagulation factors VII, VIII, IX and plasminogen activator inhibitor-1, which can promote thrombosis. Therefore, the above indexes can be used as predictors and auxiliary diagnostic indexes for the formation of DVT (35).

Venous stasis, hypercoagulable state and intimal injury are the three basic factors for the formation of DVT. Any disease or clinical condition that can cause the above abnormalities can induce thrombosis, including primary factors such as genetic variation, as well as secondary factors such as surgery, trauma, underlying diseases, immobilization and so on (36). Diabetes, chronic kidney disease and atrial fibrillation are common diseases in clinic, which often lead to secondary lesions of blood vessels and cause the blood in a hypercoagulable state (37). For example, hyperglycemia, hyperinsulinemia and insulin resistance in patients with diabetes can directly damage endothelial cells and endothelial function, and can cause chronic inflammation and participate in the process of endothelial injury. At the same time, the blood flow in the limbs of patients with diabetes is lower than that of normal people, and severe hyperglycemia can accelerate the state of hypercoagulability (38). In addition, spinal fractures, brain trauma, thoracic trauma, and the severity of SCI will all affect the occurrence of DVT. For example, complete SCI, especially SCI above T6, will seriously affect the sympathetic control of cardiac function, resulting in decreased myocardial contractility and dilatation of capillaries, gastrointestinal vascular beds and coronary arteries, resulting in a reduction of effective blood volume by about 50%. This not only increases the blood viscosity, but also greatly reduces the oxygen supply of muscle and the contractility of muscle, which eventually leads to the decrease of deep venous blood flow velocity of lower extremities and increases the incidence of DVT (39). In addition to the effects of the complications themselves, these complications also prolong the patient's bed rest and braking time. After lying in bed for a long time, the venous blood flow of the lower extremities slows down or even stagnates, and the slow flow leads the valve in a state of hypoxia, causing endothelial injury. Blood stasis can also cause the accumulation of local coagulation factors and the consumption of inhibitory factors, resulting in the formation of thrombus in the vein (40).

While several factors such as baseline characteristics, biochemical indicators, and injury severity can contribute to the development of DVT in patients with SCI, it is important to note that not all patients are affected by these factors to the same degree. Furthermore, the impact of various risk factors on DVT can vary among patients. In conclusion, there are significant differences in the risk of DVT among different SCI patients. As reported by Chibbaro et al., patients in different risk strata responded differently to the same thromboprophylaxis (41). Therefore, early identification and classification of patients in different risk strata is crucial. Our study reveals that patients with SCI face 24 risk factors that heighten the risk of DVT. Notably, the severity of SCI, smoking, diabetes, chronic kidney disease, small-intestinal bacterial overgrowth, and time from injury to admission increase the risk of DVT by more than double. These six factors have a significantly greater impact on the occurrence of DVT compared to the remaining 18 risk factors. It is recommended that clinicians and patients give greater consideration to these noteworthy risk factors. Of course, identification and prevention of other risk factors are also important. In addition, our meta-analysis revealed that the occurrence of DVT was twice as high in patients with acute SCI compared to those with chronic SCI, which is in line with previous research studies. Most DVT occurs in the first three months of SCI (acute phase), and its peak occurs from the 7th day to the 14th day after injury (42, 43). The main reason is that as time progresses, the degree of vascular intima damage and blood stasis are gradually reduced, the function of autonomic nerves is also gradually restored, and the regulation of the blood coagulation system is gradually strengthened, resulting in a gradual decrease in the incidence of DVT in the chronic phase (44–46). Therefore, it is very important to take preventive measures as soon as possible to reduce the incidence and severity of DVT after SCI.

It is worth noting that although we get the risk factors of DVT based on the data of multivariate analysis, DVT is not caused by only one factor, and the prevention of DVT should also be a comprehensive prevention in many aspects. At present, the recognized comprehensive prevention strategy requires health education, combined drug therapy and physiotherapy. Health education should include disease education (risk factors, etiology, inducement, symptoms, signs, consequences), diet education (avoid alcohol and tobacco, avoid high-fat and high-salt diet, drink plenty of water appropriately), physical activity education (early physical activity and correct physical activity method) and so on. In the past, anticoagulants such as low molecular weight heparin and warfarin were widely used for early prevention of DVT. Although the effectiveness of these drugs has been proved, the risk of spinal epidural and subdural hemorrhage increases while preventing DVT, and the hematoma may oppress the spinal cord and aggravate the SCI (47). Therefore, patients with SCI are cautiously advised to use anticoagulants, mainly using physical prophylaxis such as drug prophylaxis combined with air pressure therapy and wearing elastic socks, so as to minimize the adverse reactions caused by drugs (9, 48). In short, our study found that the incidence of DVT after SCI is high, there are many risk factors. Clinicians and patients are clearly aware of the current situation of DVT and take preventive measures as soon as possible.

### 4.2. Limitations

Data sources and analysis methods for the meta-analysis were influenced by the reporting methods of the included studies. ① The included study did not clearly report the severity of SCI (AIS grades A–E), the time of injury (from acute to chronic) and the location of injury (from cervical spinal cord to lumbosacral spinal cord). ② Details such as baseline characteristics, whether blinding was performed, and follow-up of patients were not clearly reported. ③ The treatment of patients with SCI was not reported in detail. It is worth noting that the prevention programs in different studies are significantly different, which may affect the incidence and reporting rate of DVT, thus affecting the reliability of meta-analysis results (49). These problems are the aspects that should be improved and enhanced in future research: to further refine the incidence of DVT in patients with different characteristics of SCI in order to provide more accurate information. The above limitations of the inclusion study also make it impossible for us to conduct further subgroup analysis to provide more information. In addition, even if we collected all the studies on DVT after SCI as much as possible, we did not search the grey database and excluded non-English literature, resulting in a certain publication bias and language bias, which is also proved by the asymmetric funnel plot.

## 5. Conclusions

DVT increases the disease burden of patients with SCI. However, there is a lack of large sample, multicenter epidemiological evidence. As the first study, having analyzed 101 studies including 223,221 patients, we found that the incidence of DVT in patients with SCI was 9.3% and that the incidence of DVT decreased gradually with the accumulation of publication years and sample size, but the annual incidence of DVT increased gradually from 2017. There are 24 kinds of risk factors that may contribute to the formation of DVT, involving multiple aspects of the baseline characteristics of the patient, biochemical indicators, severity of SCI, and comorbidities. Our results provide effective evidence for taking measures to prevent DVT as early as possible.

## Data Availability

The original contributions presented in the study are included in the article, further inquiries can be directed to the corresponding author.
